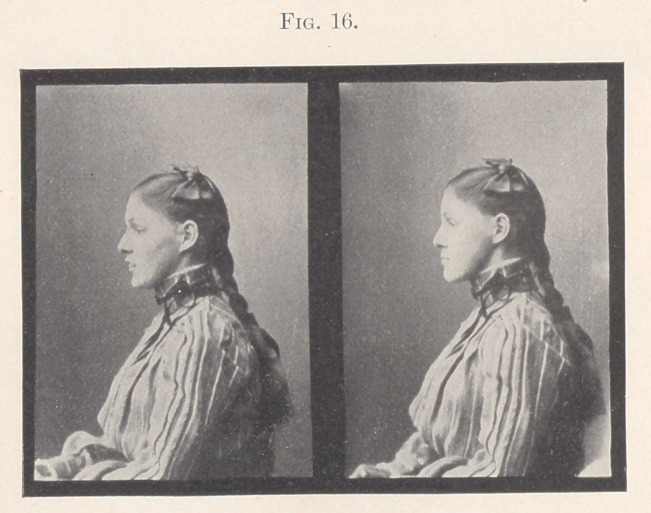# Distal and Mesial Occlusion: Some Causes and Some Results

**Published:** 1905-03

**Authors:** Horace L. Howe

**Affiliations:** Boston, Mass.


### International Dental Journal.

Vol. XXVI. MARCH, 1905. No. 3.

### Original Communications.[1]

### DISTAL AND MESIAL OCCLUSION: SOME CAUSES AND SOME RESULTS.

BY HORACE L. HOWE, D.M.D., BOSTON, MASS.

It is an undisputed fact that of all the different classes of malocclusion of the teeth, the two forms which tend to mar the facial expression the most are distal and mesial occlusion.

The open mouth, the protruding upper teeth, the undeveloped nose, and vacant expression of the mouth-breather with distal occlusion is a sight familiar to us all. Hardly less familiar is the bull-dog expression of the prognathous lower jaw with mesial occlusion.

Admitting that the face is the mirror of the soul, hence the outward expression of the mental processes, it becomes of especial interest to us as dentists to recognize and to study the abnormal conditions which tend to distort the normal facial lines, and, if possible, to rectify the malocclusion; thus enabling the person to breathe normally, to masticate food perfectly, and at the same time improving the powers of expression both by voice and by the countenance.

[1] The editor and publishers are not responsible for the views of authors of papers published in this department, nor for any claim to novelty, or otherwise, that may be made by them. No papers will be received for this department that have appeared in any other journal published in the country.

To avoid confusion, let us first consider distal occlusion. This condition is nearly always associated with mouth-breathing, which is usually first caused by adenoid growths in the nasopharyngeal vault. These growths are most commonly seen in the North Temperate Zone, and occur from infancy to puberty. Their cause is conceded to be due to an hypertrophy of the tissues brought on by inflammatory changes resulting from colds. In most cases they disappear at puberty, largely due to a diminution of the morbid activity of the tissues.

If these growths do not disappear in childhood either naturally or by surgical operation, the person will continue to breathe through his mouth; first, because of the physiological law, "That the use of a part tends to stimulate its growth and development, and disuse to lack of development and atrophy."

The nose, unused, becomes undeveloped and blocked. Second, the relaxed state of the muscles of the lips, and the abnormal pressure of the muscles of the cheeks, together with lack of occlusal support, causes the contraction and protrusion of the upper arch and the recession and distal occlusion of the lower arch. Consequently, the child in this state will breathe through the mouth because it is easier and of his inability to close his lips over the protruding front teeth.

Again, distal occlusion may be caused by some seemingly slight habit of the child when very young, such as sucking the thumb and the rubber tubes and nipples of nursing-bottles.

These are undoubtedly great factors in causing the deformity. Is it not reasonable to suppose that the lower jaw might be pushed backward in its socket by a heavy nursing-bottle hanging from it and left there for hours at a time, especially when we consider how much the flexible bones of babyhood may be changed?

For instance, the abnormal shapes of the skulls of the ancient Peruvians and Flat-Head Indians were caused by simply bandaging the heads of their children when very young.

Lastly, when there is a decided irregularity anterior to the sixth-year molar, the premature extraction of the temporary teeth accounts for this type of distal occlusion. Mouth-breathing does not usually accompany this type of distal occlusion.

To illustrate this form of distal occlusion, let us consider Fig. 1. This slide was made from a specimen obtained from the Warren Museum of the Harvard Medical School. The subject was evidently

Fig. 1.

Fig. 2.

Fig. 3.

Fig. 4.

FIG. 5.

FIG. 6.

a child about six years old. All of the temporary teeth, excepting the two inferior centrals, are in position; the sixth-year molars of the mandible have erupted, while those of the maxillæ have partly erupted. The permanent teeth are beautifully shown partially formed, and which will succeed the temporary ones in their normal positions, providing nothing is done to disturb their course of eruption. But we can easily understand, if the second temporary molar was extracted prematurely, the sixth-year molar would erupt forward of its normal position, and its distal buccal cusp would engage in the buccal groove of the lower molar instead of its mesial buccal cusp. This necessarily would cause an irregularity anteriorly.

Case I.—Fig. 2 shows a typical case of this kind. The right second superior temporary molar was extracted two years prematurely. The sixth-year molar erupted forward in its place, causing the second bicuspid to erupt out of line, as is shown by the palatal view. Upon the left side the condition is better, but owing to the badly decayed second temporary molar, the sixth-year molar is a trifle mesially dislocated.

Case II.—The models shown in Fig. 3 represent a case with typical distal occlusion. The patient, a boy of fourteen, had the habit of sucking his lower lip almost constantly. Probably adenoids were formerly associated with the case. You will notice that every tooth of the lower arch occludes *distally* instead of *mesially* to its corresponding fellow of the upper arch. The first lower molar, for instance, occludes distally to the upper first molar, and so on.

Fig. 4 presents the case three months from the time the previous models were made. Normal occlusion is established, with the result that the facial lines are improved and the boy can breathe normally through his nose.

Fig. 5 shows the palatal view of the two previous models compared.

Fig. 6 shows the photographs compared.

Case III.—Fig. 7 illustrates another case of typical distal occlusion. Patient twelve years of age. Notice, if you will, the remains of the right, second temporary molar.

Fig. 8 shows normal occlusion established with the exception that the second right upper bicuspid has not fully erupted.

Fig. 9 is a profile view showing difference in facial lines. Observe, if you please, that the shape of the nose is even changed. The drawn expression and hollow cheeks have disappeared, all the change taking place in six months' time.

Fig. 10 shows a front view of the photographs compared. This case illustrates the advantage of correcting the malocclusion as soon as possible after being recognized, that the face may develop along normal lines as the child grows older, instead of along abnormal lines.

CASE IV.—Fig. 11 is a very interesting case of a child three and a half years of age. This case goes to confirm the theory of Dr. Henry Baker that mesial occlusion is often caused by habit. This child had the habit of protruding the lower jaw forward so that the lower front teeth would lock in front of the upper incisors. The jaw became accommodated to its new position, so that the child was unable to bite normally. Her family physician noticed the deformity and remarked upon it. She came under my observation, and I instructed the parents of the child to tell her to bite back as much as possible. The result was that within two months the child was able to bite normally, as is shown by the model upon the left.

The tendency to mesial occlusion was undoubtedly inherited, as both parents have slight mesial occlusion. This case, with others, convinces me that mesial occlusion is usually caused by the jaw being too far forward in its socket, rather than by any abnormal development of the jawbone itself.

CASE V.—Fig. 12 shows a case of mesial occlusion. The patient is a man twenty-eight years old, who when young had the habit of protruding the lower jaw forward and sucking his fingers with the tips behind the lower incisors. In this case the patient was unable to project his jaw any farther forward than he usually held it.

Fig. 13 shows the improved occlusion, although not perfect. This condition was obtained without grinding the back teeth at all. The condition is improving constantly rather than growing worse, as the case would have been providing nothing was done. The facial lines show a marked improvement, although I have not the privilege of showing photographs.

I am indebted to Dr. W. H. Parker for the privilege of showing the following case. The patient, a girl of fourteen, recently came to him for treatment with the teeth in the condition that we see them in Fig. 14, caused, the mother said, by the child's sucking and pulling upon the rubber tube of a nursing-bottle. Three years ago she was evidently in the hands of an enthusiastic extractor, as

Fig. 7.

Fig. 8.

Fig. 9.

Fig. 10.

Fig. 11.

Fig. 12.

Fig. 13.

Fig. 14.

Fig. 15.

Fig. 16.

the four perfectly sound sixth-year molars were removed to correct the protruding front teeth. The dismal failure we can all see.

The remarkable feature of the case is that a year ago the child was told that if she would close her lips it would improve her appearance. She bravely made the attempt, and protruded the lower jaw forward so that the teeth occluded, as we see them in Fig. 15, thus enabling her to close her lips. The girl therefore came to have practically two bites,—the old bite, to which she was forced to return for masticating, and the forward bite, which enabled her to close her lips and to breathe normally.

Incidentally, the mother stated that the child could endure having her teeth regulated now better than she could a year ago, as her health was much better.

But in the attempt to conceal one deformity the child developed another, not as marked, perhaps; still the facial expression lacks harmony, as we can all see by Fig. 16. The facial expression of the picture upon our right is that of a typical prognathous lower jaw, it being caused by her extreme forward bite.

From these cases we are able to draw several conclusions. First, *that distal occlusion* may be caused by the lower jaw being forced backward in its socket by the same means that force the upper teeth forward.

Second, *that the improvement in general health* which invariably follows the establishment of normal occlusion is not accidental, but due to normal breathing through the nose, improved nutrition resulting from better mastication of food, and because the stomach is less contaminated by the foul secretions of the unused nose.

Third, *that the muscles, the condyles, and the teeth* of the lower jaw will accommodate themselves to new positions of the jaw. This is especially true if the teeth of the upper arch are made to give occlusal support to the teeth of the lower jaw in its new position.

In a previous part of this paper I stated that it becomes of especial interest to us as dentists to recognize and to study these deformities, and, if possible, to rectify the malocclusion, thus enabling the person to breathe normally, to masticate food perfectly, and at the same time improving the powers of expression both by voice and by countenance.

We have reason to be proud of the fact that a Boston man, Dr. Henry A. Baker, has given to the profession the intermaxillary elastics which afford a practical method for the correction of these cases. Dr. E. H. Angle in a recent article said, "The Baker anchorage has revolutionized the practice of orthodontia." I can say no more in appreciation of their value than to simply indorse this statement.

Finally, the results of distal occlusion with consequent mouth-breathing may be far reaching. Breathing through the mouth from necessity is shallow breathing. The air is drawn into the lungs without being filtered, warmed, or moistened. From lack of deep breathing the lungs become small and contracted, and the person hollow-chested. The blood is not properly aërated. The whole vitality is lowered, and the powers of resisting diseases are impaired, hence there is a greater susceptibility to all diseases, especially diseases of the air-passages.

Altogether the mouth breather is poorly equipped for life's work. To have a perfect working machine, the most minute parts are looked after and kept in order. If one tiny wheel goes wrong, it impairs the total work of the machine. If not set aright, it will be but a short time before the whole machine is useless. Why not as true in the case of the human machine, the most wonderfully constructed of them all?

The influence of health upon the career of the individual is aptly shown by John Tyndall in his address to students, where he says, "Take care of your health. There have been men who by wise attention to this point might have risen to any eminence,—might have made great discoveries, written great poems, commanded armies, or ruled states, but who by unwise neglect of this point have come to nothing. Imagine Hercules as oarsman in a rotten boat; what can he do there but by the very force of his stroke expedite the ruin of his craft? Take care, then, of the timbers of your boat, and avoid all practices likely to introduce either wet or dry rot among them. And this is not to be accomplished by desultory or intermittent efforts of the will, but by the formation of habits. The will, no doubt, has sometimes to put forth its strength in order to strangle or crush the special temptation. But the formation of right habits is essential to your permanent security. They diminish your chance of failing when assailed, and they augment your chance of recovery when overthrown."

It is unnecessary perhaps to say in conclusion that habits which impair the vital functions of nutrition and respiration must influence the health and hence the career of the individual.

## Figures and Tables

**Fig. 1. f1:**
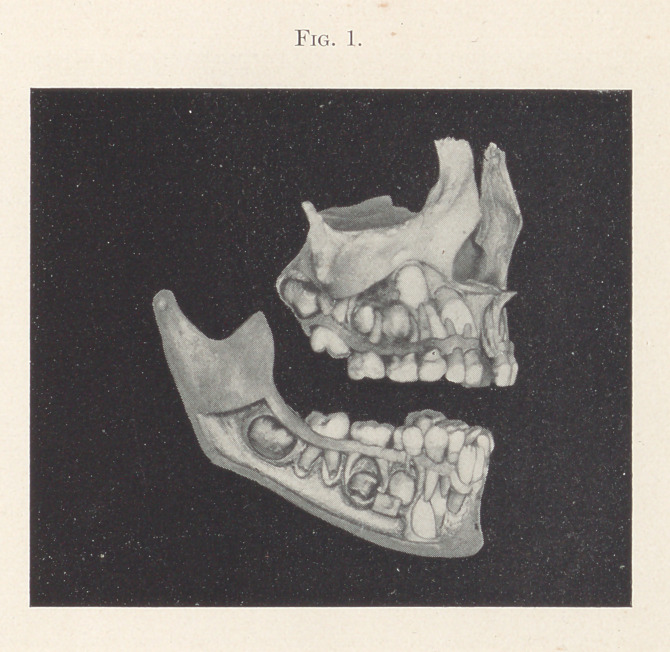


**Fig. 2. f2:**
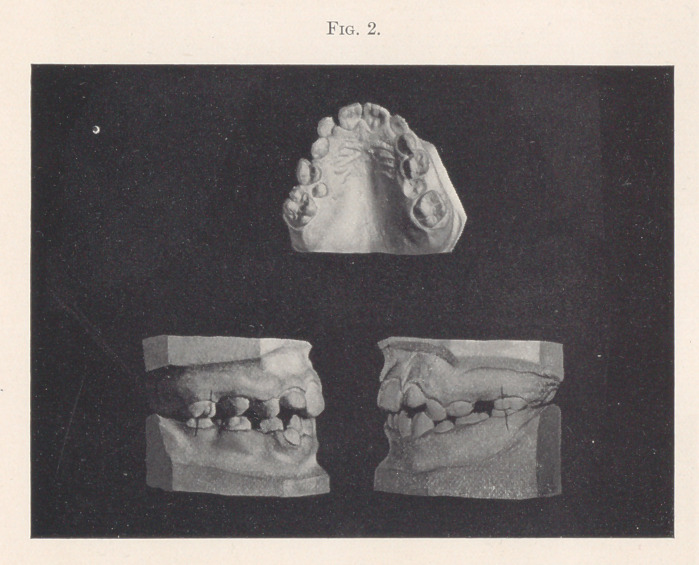


**Fig. 3. f3:**
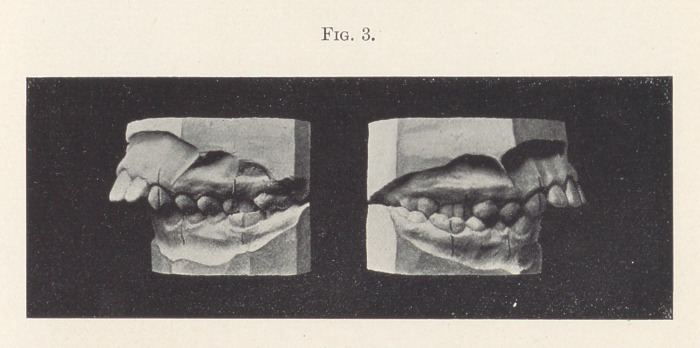


**Fig. 4. f4:**
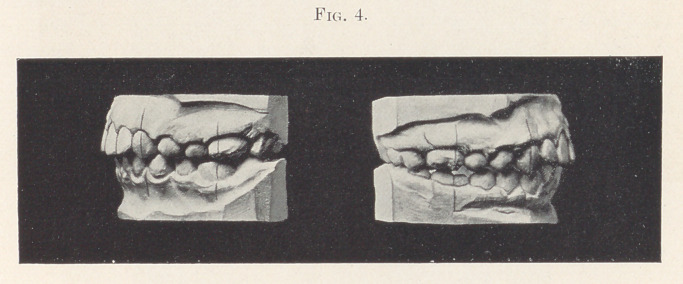


**Fig. 5. f5:**
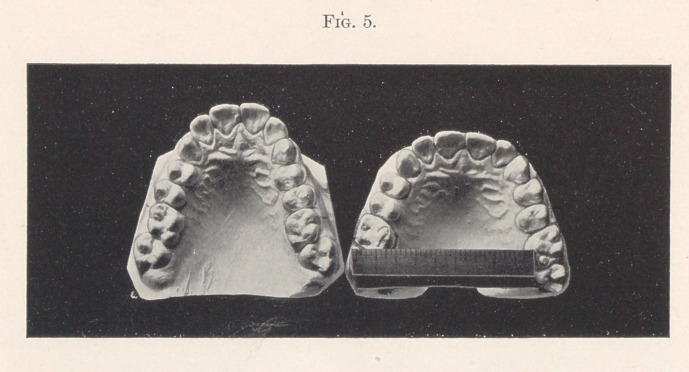


**Fig. 6. f6:**
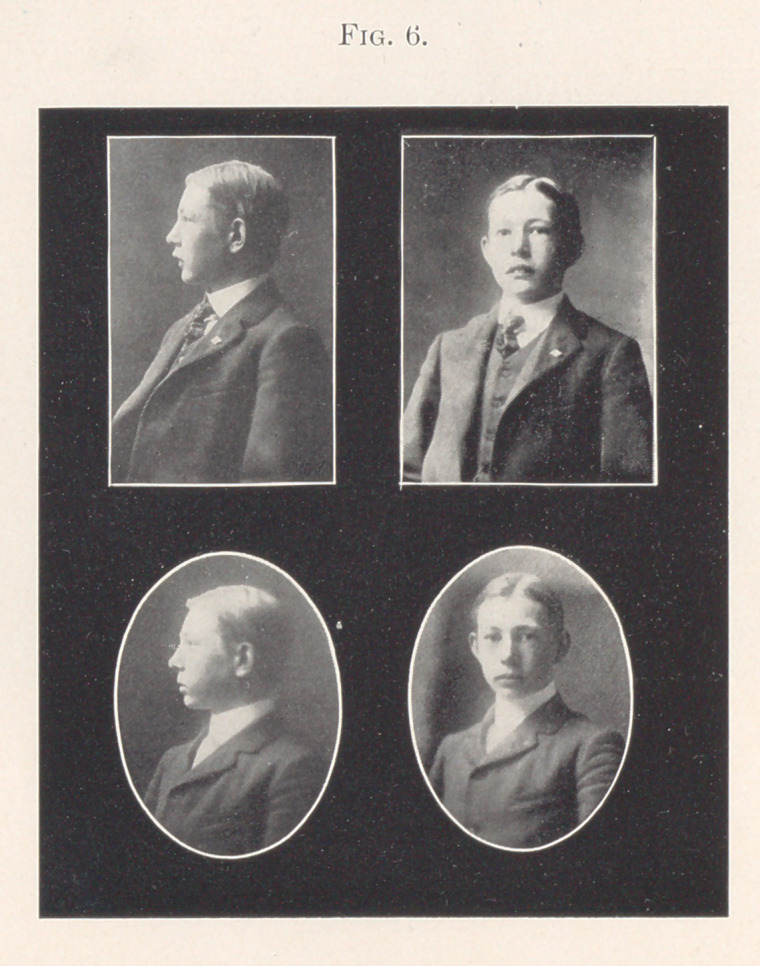


**Fig. 7. f7:**
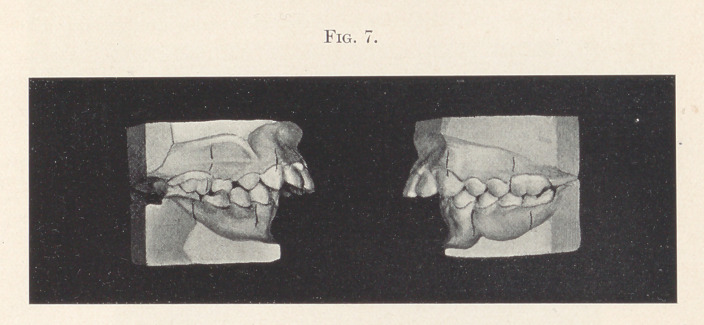


**Fig. 8. f8:**
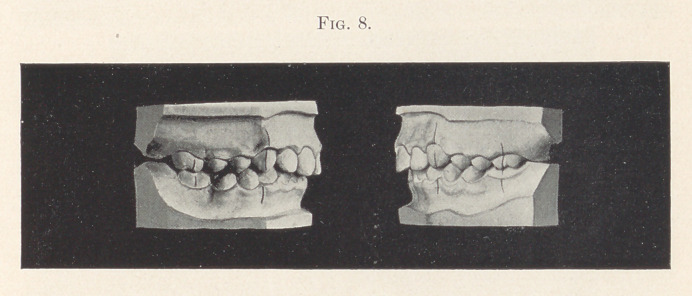


**Fig. 9. f9:**
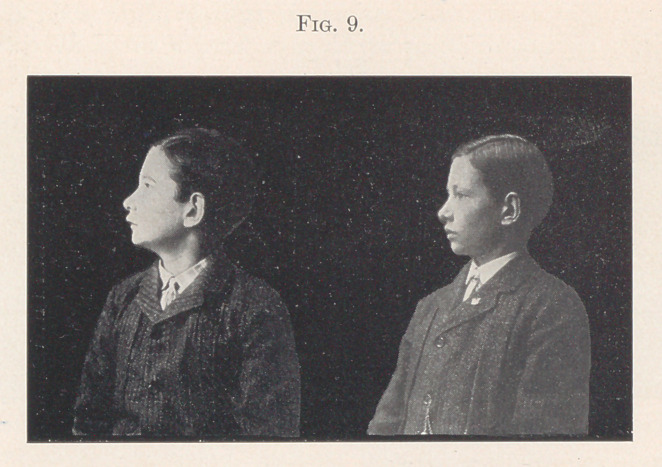


**Fig. 10. f10:**
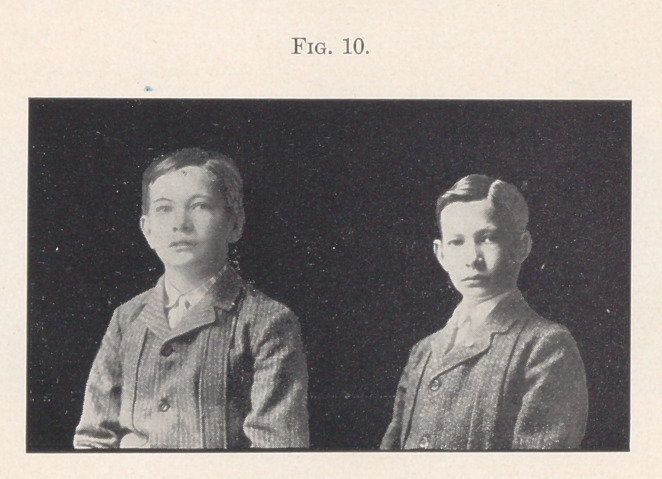


**Fig. 11. f11:**
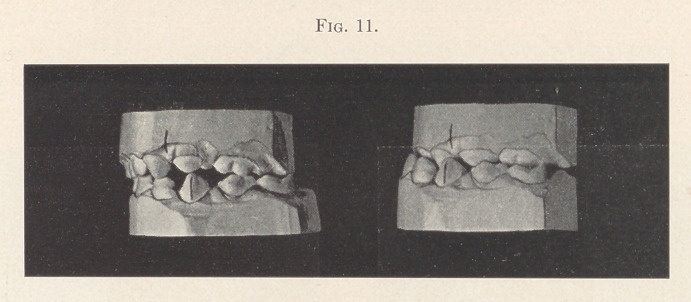


**Fig. 12. f12:**
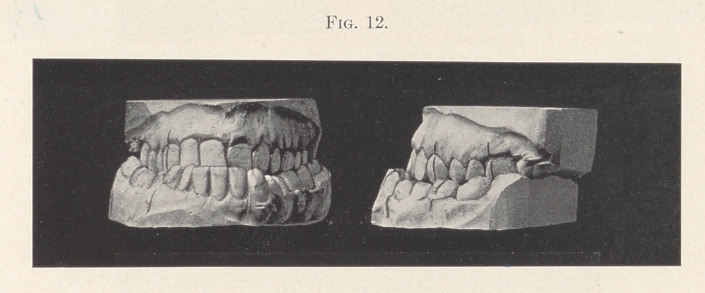


**Fig. 13. f13:**
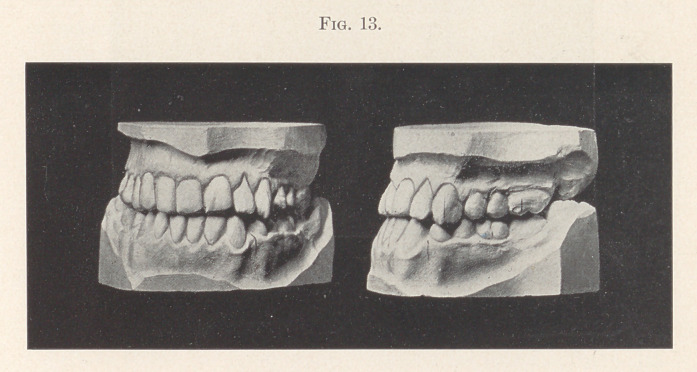


**Fig. 14. f14:**
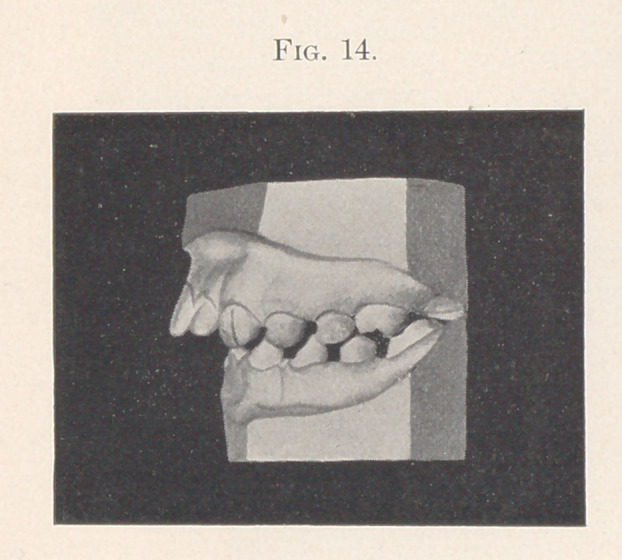


**Fig. 15. f15:**
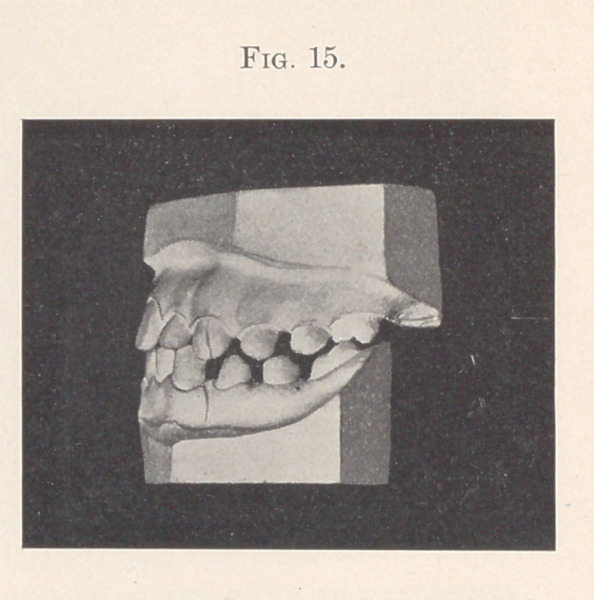


**Fig. 16. f16:**